# Ultrasound-guided intra-muscular botulinum toxin A in athletes with chronic adductor-related groin-pain: A retrospective observational study

**DOI:** 10.1016/j.jsampl.2025.100105

**Published:** 2025-06-04

**Authors:** Julien Orhan, Romain Garofoli, Émilie Alperin, Fabien Ladauge, Jennifer Zauderer, Guillaume Paris, François Rannou, Christelle Nguyen, Marie-Martine Lefèvre-Colau

**Affiliations:** a AP-HP.Centre-Université Paris Cité, Service de Rééducation et de Réadaptation de l’Appareil Locomoteur et des Pathologies du Rachis, Hôpital Cochin, 75014, Paris, France; b AP-HP.Centre-Université Paris Cité, Service de Radiologie Ostéo-Articulaire, Hôpital Cochin, 75014, Paris, France; c Université Paris Cité, Faculté de Santé, UFR de Médecine, 75006, Paris, France; d INSERM UMR-S 1124, Toxicité Environnementale, Cibles Thérapeutiques, Signalisation Cellulaire et Biomarqueurs (T3S), Campus Saint-Germain-des-Prés, 75006, Paris, France; e ECaMO Team, INSERM UMR-S 1153, 75004, Paris, France

**Keywords:** Chronic athletic pubalgia, Botulinum toxin, Groin pain, Adductor enthesopathy

## Abstract

**Introduction:**

Chronic adductor-related groin pain (AP) is a frequent and disabling sport condition. Intra-muscular injection of botulinum toxin A may have positive effects on pain in some chronic tendinitis. We aimed to describe the short-term evolution of pain, activity limitations and quality of life, after an injection of the *adductor longus* with botulinum toxin A, as an add-on therapy to standard of care in patients with chronic AP.

**Method:**

We conducted a retrospective observational single-centered study. We included individuals with clinical and MRI chronic AP, for whom medical and/or surgical treatments have failed and who were treated with an intra-muscular injection of botulinum toxin A (100 units of botulinum toxin A in the *adductor longus*) under ultrasound guidance. Participants were assessed 50 days after injection for pain using a numerical rating scale (NRS) and for activity limitations and quality of life using the Copenhagen Hip and Groin Outcome Score (HAGOS). Participants were also asked to self-report adverse events.

**Results:**

We included 20 participants. Mean age was 34.3 (11.7) years and mean symptom duration was 48.9 (61.6) months. Mean pain decreased from 55.3 (SD [22.4] before injection to 38.3 [21.7], 50 days after injection (*p* ​= ​0.027). Each of the 6 HAGOS subscales improved before and after injection. No serious adverse events were self-reported by the patients included in the main analysis.

**Conclusion:**

In this retrospective uncontrolled trial, we observed a numerical decrease in pain intensity in individuals with chronic AP 50 days after intra-muscular botulinum toxin A injection in the *adductor longus*.

## 1 Introduction

Chronic groin pain (GP) in athletes is a painful syndrome located in the inguinal area, lasting more than 3 months.

The Doha Agreement Meeting on terminology and definitions of GP in athletes divided GP into three categories: 1. Defined clinical entities for groin pain: adductor-related (AP), iliopsoas-related, inguinal-related and pubic-related GP, 2. Hip-related GP, 3. Other causes of GP in athletes [1]. As iliopsoas-related GP referred to hip-related GP and as enthesopathy of the rectus abdominis has been recently described [2], some authors considered that it can be divided into 4 causes: the enthesopathy of the *adductor longus*, the enthesopathy of the rectus abdominis (corresponding to the adductor-related groin pain), the pain from the pubic symphysis (corresponding to the pubic-related groin pain), and the pain induced by a defect on the posterior and anterior sides of the inguinal canal (corresponding to the inguinal-related groin pain) [3]. These definitions and terminology are based on history and physical examination along with Doha agreement and need to rule out hip-related groin pain and other causes of groin pain in athletes such as inguinal or femoral hernia, nerve entrapment, referred pain from the lumbar spine, the sacroiliac joint, apophysitis or avulsion fracture [1].

Chronic athletic GP is diagnosed in athletes complaining of GP, unilateral in 60 ​% of cases and often recurrent, and can last for several months or even years due to diagnostic delay [3]. The main clinical signs are inguinal and/or abdominal pain exacerbated during specific sports activities such as sprinting, kicking, doing sit-ups, and being relieved by rest. Chronic athletic GP affects 5 ​%–18 ​% of athletes in all sports [3,4]. Some studies showed that up to 58 ​% of footballers have a history of pubic pain and that 50 ​% of chronic athletic GP persists more than 20 weeks after the first symptoms [5,6]. Chronic athletic GP also occurs more frequently in men; only 10 ​% of patients with GP are women [3,7]. The two muscles most frequently affected are the *adductor longus* in 58 ​% of cases and the rectus abdominis in 27 ​% of cases. Mixed forms exist in 40 ​%–80 ​% of cases [3].

The first-line treatment of GP is conservative and targets pain. It consists of oral non-steroidal anti-inflammatory drugs, associated or not with corticosteroid injections, and physical therapy for 2–6 months [3]. In case of failure of a well-conducted conservative treatment, surgery can be offered depending on the patient’s sporting plan [3]. Adductor enthesopathy is the most common form of GP, with a prevalence of 44 ​%–60 ​%, and conservative treatment has been poorly evaluated and seems to be moderately effective [8,3,9,10,11,12,13]. In a systematic review of 11 open studies (*n* ​= ​514), the return to previous habitual activity was 90 ​% with tenotomy and 80 ​% with conservative treatment. Further, 90 ​% of patients had no longer pain on a median follow-up between 18 and 26 months compared to 67 ​% in with conservative treatment [9]. Recently, intra-muscular botulinum toxin A has raised interest because it may have positive effects on pain in some chronic tendinopathies such as lateral epicondylitis [14,15,16,6,17,18]. Wong et al. showed in their study a decrease in pain, from 65.5 ​mm to 25.3 ​mm, four weeks after injection in patients with lateral epicondylitis treated with botulinum toxin A [18].

We hypothesized that similar analgesic effect of intra-muscular botulinum toxin A may be observed in individuals with chronic AP. In the present study, we aimed to describe the short-term evolution of pain and of activity limitations and quality of life, after an injection of the *adductor longus* with botulinum toxin A, in individuals with chronic AP, for whom medical and/or surgical treatments have failed. This particular muscle was chosen because AP is the most common form of chronic athletic GP and the adductor longus the most frequently affected muscle [3].

## 2 Methods

### 2.1 Design

We conducted a retrospective observational single-centered study. Our study is reported in accordance with the Strengthening the Reporting of Observational Studies in Epidemiology (STROBE) statement [19] **(E-component 1)**.

### 2.2 Participants

**Fig. 1:**
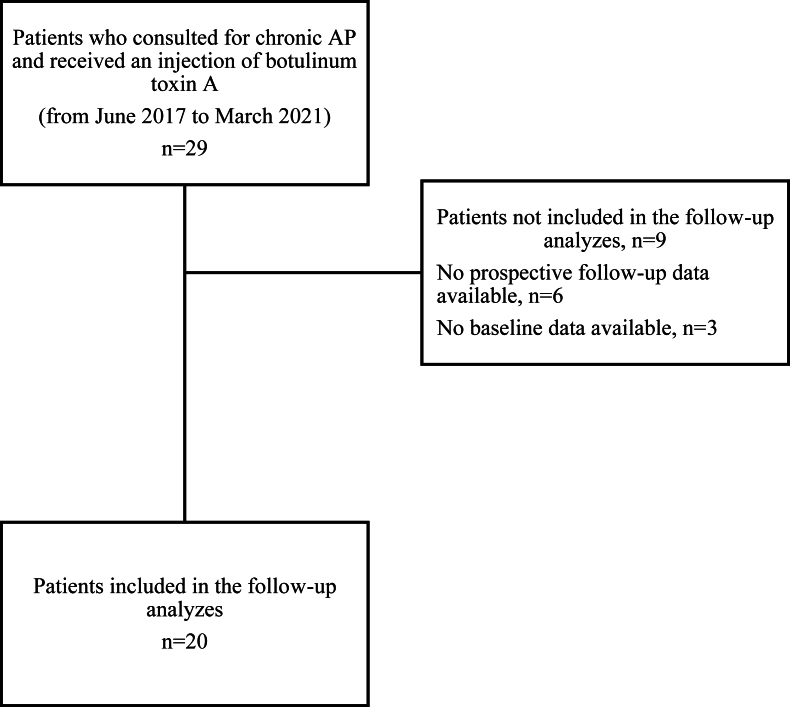
Flow diagram.

Individuals referred between June 2017 and March 2021 to a consultation of physical medicine and rehabilitation at Cochin hospital of a single physician (GP) were retrospectively and consecutively screened. Inclusion criteria were: 1/chronic adductor-related groin pain (AP), 2/symptom duration ≥3 months, 3/consistent clinical and magnetic resonance imaging findings of enthesopathy of the *adductor longus* according to the clinician expertise, 4/failure of medical and/or surgical treatments with persistent pain and inability to return to play, and 5/treatment with an intra-muscular injection of botulinum toxin A injection. Exclusion criteria were: 1/age under 18 years, 2/ongoing pregnancy or breastfeeding, 3/treatments by anticoagulants, 4/muscular disorder (e.g. polymyositis, myopathy or other neuromuscular conditions), 5/guardianship or curatorship, and 6/individuals who had received botulinum toxin A treatment for less than one year were not included. Individuals for whom pain intensity and/or Copenhagen Hip and Groin Outcome Score (HAGOS, 0, worse outcome, and 100, most favorable outcome) at baseline and/or follow-up were not available, were not included in analyses, and are reported in the flow diagram (Fig. 1).

### 2.3 Interventions

Botulinum toxin A injection was performed by the same treating physician (GP) under ultrasound guidance. The 100 units dose of botulinum toxin A (Xeomin®, Merz) was injected in 2 muscular locations in the *adductor longus* on the affected lower limb. Ultrasonography was performed with a linear high frequency probe (20 Mhz) on a patient in supine position. Our experimental intervention design was based on the dose and the injection sites commonly used for treating *adductor longus* spasticity in neurological disorders: injections were performed 35 ​% and 50 ​% from anterior superior iliac spine [20]. Following the injection, participants were instructed to observe a relative rest period of 3–4 days and were prescribed to start rehabilitation thereafter supervised by a physiotherapist, according to the ASPETAR protocol [21]. Two patients received only 50 and 40 UI in the *adductor longus* at the beginning of the protocol but we decided to include them in the analyses as the only risk to include them would have been to decrease the positive effect of the botulinum toxin A injection. All pharmacological and non-pharmacological co-interventions were allowed during follow-up, as prescribed by the treating physician, but were not recorded.

### 2.4 Outcomes

Baseline characteristics of participants and pain numerical rating scale (NRS, 0, no pain, and 100, maximal pain) were collected by the treating physician (GP) or some authors (JO, RG, FL) on the day of injection. Magnetic resonance imaging (MRI) was assessed by a board-certified radiologist (ÉA) using a standardized checklist for the presence of the following items: 1/congestive tendinopathy, 2/location of congestive oedema (i.e., ischiopubic branch, adductor enthesis, *abdominus longus* enthesis) and 3/congestive pubic arthropathy. The HAGOS was calculated using a self-administered questionnaire [22,23]. Briefly, the HAGOS assesses the clinical course of pubalgia, in particular daily and sport activity limitations. The scale consists of six subscales (associated symptoms, pain, limitations in activities of daily life, limitations in sport activities and quality of life) for active patients (>2.5 ​h per week). For the follow-up, participants were contacted by one of the authors (JO) by phone, approximately 50 days after injection (median ​= ​30 days [30.0–47.3], min ​= ​30 days, max ​= ​203 days) to collect pain NRS and HAGOS. Participants were also asked to rate their improvement using a 3-class scale (i.e., totally improved, partially improved or not improved at all) and to self-report any adverse events.

### 2.5 Statistical analyses

Descriptive analyses were carried out using Excel software. Quantitative data were described with mean (standard deviation [SD]) and qualitative data with absolute and relative frequencies. The comparative data analysis of pain before and after injection was carried out by the biostaTGV online software (http://biostatgv.sentiweb.fr/) using the Wilcoxon-Mann Whitney test. The test was considered significant when the p value was less than 0.05.

### 2.6 Funding source and ethical consideration

Our study was not funded. It was approved by our Institutional Review Board (CERAPHP, n° 00011928) on February 17, 2021. All participants were informed and gave written consent to participate.

## 3 Results

### 3.1 Participants

From June 2017 and March 2021, 29 individuals were screened: 6 were excluded and 3 could not be included in the analyses because of missing data. Overall, 20 eligible individuals were included in the analyses (Fig. 1). Mean age was 34.3 (11.7) years, mean symptom duration was 48.9 (61.6) months and 2/20 (10 ​%) were women. Sports involved were football in 9/20 (45 ​%) participants and running in 3/20 (15 ​%). Eleven (55 ​%) participants had surgery prior to botulinum toxin A injection. Out of 20 patients, we were able to review only 12 MRI: among them, seven participants had a congestive tendinopathy on magnetic resonance imaging (Table 1a) (see Table 1b).

**Table 1a:** Characteristics at baseline of patients included in the follow-up analyzes.

Population (*N* ​= ​20)	
Age (years), mean (SD)	34.3 (11.7)
Female, n (%)	2 (10)
Symptom duration (months), mean (SD)	48.9 (61.6)
Sport, n (%)	
Football	9 (45)
Running	3 (15)
Dance	1 (5)
Badminton	1 (5)
Horse riding	1 (5)
Boxing	1 (5)
Judo	1 (5)
Triathlon	1 (5)
Rugby	1 (5)
None	1 (5)
Magnetic resonance imaging findings, n (%)	
Patients with MRI reviewed	12/20 (60)
Congestive tendinopathy	7 (58.3)
Location of congestive oedema	
Ischiopubic branch	2 (16.7)
Adductor enthesis	6 (50)
*Abdominus longus* enthesis	0 (0)
Congestive pubic arthropathy	1 (8.3)
Previous treatments, n (%)	
Corticosteroid injection	3 (15)
Physical therapy	20 (100)
Past history of surgery	11 (55)
Adductor tenotomy	10 (50)
Shouldice	3 (15)
Shouldice and adductor tenotomy	2 (10)
Mean time elapsed between injection and follow-up, days (SD)	51.5 (50.6)

n ​= ​number, SD ​= ​standard deviation.

There are no missing data.

**Table 1b:** Characteristics at baseline of patients not included in the follow-up analyzes.

Population (*N* ​= ​9)	
Age (years), mean (SD)	30.4 (9.0)
Female, n (%)	0 (0)
Symptom duration^a^ (months), mean (SD)	26.6 (25.1)
Sport^b^, n (%)	
Football	5 (56)
Running	1 (11)
Boxing	1 (11)
Judo	1 (11)
Previous treatments, n (%)	
Corticosteroid’s injection	4 (44)
Physical therapy	9 (100)
Past history of surgery	4 (44)
Adductor tenotomy	4 (44)
Shouldice	3 (33)
Shouldice and adductor tenotomy	4 (44)

n ​= ​number, SD ​= ​standard deviation.

a 2 missing data.

b 1 missing data.

### 3.2 Evolution of pain, activity limitations and quality of life

Mean pain decreased from 55.3 (22.4) before injection to 38.3 (21.7) 50 days after injection (*p* ​= ​0.027), which represented an absolute difference of −17.0 (19.8) or 30.7 ​%, with an a Cohen’s d effect size of 0.77. Each of the 6 HAGOS subscales improved before and after injection, with an absolute difference varying from 3.5 (26.0) for quality of life to 18 (20.0) for everyday life. Mean HAGOS total score increased from 47.0 (12.1) to 58.6 (10.7) (Table 2). Overall, 14/20 (70 ​%) participants felt partially improved and 6/20 not improved (30 ​%).

**Table 2:** Evolution of pain and of activity limitations and quality of life after intra-muscular botulinum toxin A.

	Before injection	After injection	Absolute difference	CI95 ​%	p-value
Pain intensity^a^ (NRS, 0–100)	55.3 (22.4)	38.3 (21.7)	−17.0 (19.8)	−26.2; −7.8	0.027^c^
HAGOS^b^ (0–100)					
Everyday life	71.8 (22.8)	89.8 (9.7)	18.0 (20.0)	8.6; 27.4	–
Pain	62.6 (22.2)	76.9 (12.8)	14.3 (17.7)	5.6; 22.5	
Sport	47.8 (19.2)	61.9 (19.7)	14.1 (26.3)	1.8; 26.4	
Symptoms	58.6 (21.1)	72.5 (18.3)	13.9 (17.2)	5.8; 21.9	–
Physical activity	6.3 (11.8)	11.9 (15.4)	5.6 (11.1)	0.4; 10.8	–
Quality of life	35.3 (23.4)	38.8 (20.0)	3.5 (26.0)	−15.6; 8.6	–
Total	47.0 (12.1)	58.6 (10.7)	11.6 (11.7)	6.1; 17.0	–

HAGOS=Copenhagen Hip and Groin Outcome Score, NRS ​= ​numerical rating scale, SD ​= ​standard deviation.

All results are mean (SD). There are no missing data.

a Higher score indicates greater pain intensity.

b Higher score indicates better outcome.

c Comparisons of values before and after injection using the Wilcoxon-Mann Whitney test.

### 3.3 Adverse events

None was reported during follow-up in individual included in analyses. In individuals not included in analyses because of missing data regarding pain and/or HAGOS at baseline and/or follow-up, 3 serious adverse events were reported: one pulmonary embolism in a 35-year-old man, one surgery for GP in a 16-year-old man and one shoulder surgery in a 26-year-old man.

## 4 Discussion

In the present study, we observed a significant, clinically-meaningful numerical decrease in pain intensity in individuals with chronic adductor-related groin pain (AP), 50 days after intra-muscular botulinum toxin A injection in the *adductor longus* [24]. These results are consistent with a previous study from Creuzé et al. showing good results on pain and function 30 days (and even one year) after botulinum toxin A injection in adductor muscles, for AP [25]. In contrast with the results of the meta-analysis by Kalichman and colleagues, who reported weakness of the injected muscles, pain at the injection site and paresthesia [6], we observed no side effects in our study. One can hypothesize that other adductors could compensate the weakness of the *adductor longus* in their functional activity, and because sides effects were self-reported.

Interestingly, in this severe population suffering from AP (i.e., 55 ​% of our participants had a history of surgery), we observed that with an intramuscular injection of botulinum toxin A in the *adductor longus*, 55 ​% of participants had a significant decrease in pain intensity (30 ​% or more). In comparison, in their systematic review, Jorgensen and colleagues reported a decrease in pain measured on the NRS of 50 ​mm with conservative treatment at a median time of 10 months, and of to 216/322 (67 ​%) patients were pain free [10]. Our population was in the same range of age but was more chronic because the mean symptom duration in our study was 48.9 (61.6) months.

We observed a serious adverse effect in a 35-year-old patient who had a pulmonary embolism 5 days after the botulinum toxin A injection. After reviewing this case, we found that he was presenting clinical signs of deep venous thrombosis on the day of the injection on the same leg. Our patient was later diagnosed with anti-phospholipid syndrome. Thrombo-embolic adverse effects are rare after botulinum toxin A intramuscular injection, and little described in the literature [26,27]. Our observation suggests that clinical examination should carefully rule out deep venous thrombosis, before injecting intramuscular botulinum toxin A in the lower limb.

Our study has several limitations. First, the follow-up after toxin injection was short. Botulinum toxin A effects may last for up to 6 months. Therefore, it would have been interesting to follow-up participants up to 6 months after injection to evaluate longer-term evolution. Participants in our study had severe AP, as more than half of them had a history of surgery, and may not be representative of the general French population with chronic AP. We were able to review MRI results of only 12 out of 20 patients because of missing data. We did not record co-interventions, such as physiotherapy or pain killers intake, which may have also influenced outcomes. Nine patients had to be excluded from the analysis because they had missing data. The follow-up period was on average 50 days but it was quite different from one patient to another which is another limitation of our study. Finally, the main limitations of the study are its retrospective, monocentric and uncontrolled design and its small sample size, which do not allow drawing conclusions about the efficacy and safety of the intervention. As it is a retrospective study, the improvements observed are likely influenced by many things such as: patient expectations, regression to the mean, or concurrent rehabilitation, further underlining the need for cautious interpretation.

The definition of athletes with chronic GP in the literature varies from one paper to another and includes a wide range of pathology in this area from bone inflammation to musculotendinous lesions. Many diagnoses can be looked for when faced with an athlete complaining of GP. The diagnosis challenge is in part due to the complex anatomy of the pubis with various muscles and entheses and multiple interactions between the different structures involved. All these structures work together to allow movements and are under stress when playing some sports [28]. Treating groin pain in athletes remains complicated due to this complex anatomy and the lack of non-surgical options in the literature.

In summary, our preliminary data indicates that pain intensity numerically decreases in the short-term after intra-muscular botulinum toxin A injection in the *adductor longus* in individuals with chronic AP. Whether this observation is specific to our intervention or no needs to be further assessed in a randomized controlled trial specifically designed and dimensioned to evaluate efficacy and safety.

## Consent to participate (include appropriate statements)

All participants were informed and gave written consent to participate.

## Consent for publication (include appropriate statements)

All authors listed provided final written approval of the version to be published and agreement to be accountable for all aspects of the work in ensuring that questions related to the accuracy or integrity of any part of the work are appropriately investigated and resolved.

## Availability of data and material (data transparency)

Academic researchers can request access to data and material by contacting Dr. Marie-Martine Lefèvre-Colau at marie-martine.lefevre-colau@aphp.fr.

## Code availability (software application or custom code)

Not applicable.

## Authors' contributions

Conception and design of the study. JO, RG, CN, MMLC.

Drafting of the original protocol. JO, RG, CN, MMLC.

Coordination of the study. RG, CN, MMLC.

Acquisition of data. JO, RG, ÉA, FL, JZ, GP.

Design of the statistical analysis plan. JO, CN.

Analysis of data. JO, RG, CN, MMLC.

Drafting of the present manuscript. JO, RG, CN, MMLC.

Final approval. JO, RG, ÉA, FL, JZ, GP, FR, CN, MMLC.

## Ethical consideration

Our research was approved by our Institutional Review Board (CERAPHP, n° 00011928) on February 17, 2021.

## Additional declarations for articles in life science journals that report the results of studies involving humans and/or animals

Not applicable.

## Funding

Our study was not funded.

## Declaration of competing interest

The authors declare the following financial interests/personal relationships which may be considered as potential competing interests: Dr. Julien Orhan reported receiving advantages and hospitality from MERZ outside of the submitted work (<$5000/y). Dr. Guillaume Paris reported having been employed by MERZ and receiving advantages and hospitality from MERZ (>$30,000/y). Prof. Christelle Nguyen reported receiving consulting fees from THUASNE, speaker fees from Actelion Pharmaceuticals France, IPSEN and MEDA PHARMA, reimbursement of conference registration and accommodation by GRÜNENTHAL and MERZ, and hospitality from PRECIPHAR, Takeda France, UCB Pharma SA and SANDOZ, outside of the submitted work (<$5000/y). Other authors reported no conflict of interest.
